# Update on the Pharmacological Actions of Enoxaparin in Nonsurgical Patients

**DOI:** 10.3390/medicina60010156

**Published:** 2024-01-15

**Authors:** Egidio Imbalzano, Luana Orlando, Giuseppe Dattilo, Marianna Gigliotti De Fazio, Giuseppe Camporese, Vincenzo Russo, Alessandro Perrella, Francesca Futura Bernardi, Pierpaolo Di Micco

**Affiliations:** 1 Department of Clinical and Experimental Medicine, University of Messina, 98122 Messina, Italy; egidio.imbalzano@unime.it (E.I.); orlandoluana29@gmail.com (L.O.); giuseppe.dattilo@unime.it (G.D.); mariannagdf89@hotmail.it (M.G.D.F.); 2 General Medicine Department, Thrombotic and Haemorrhagic Disorders Unit, Department of Internal Medicine, University Hospital of Padua, 35131 Padua, Italy; giuseppe.camporese@aopd.veneto.it; 3 Department of Translational Science, University Vanvitelly, 81025 Caserta, Italy; v.p.russo@libero.it; 4 Unit Emerging Infectious Disease, Ospedali dei Colli, P.O. D. Cotugno, 80131 Naples, Italy; alex.perrel@virgilio.it; 5 Department of Experimental Medicine, Università degli Studi della Campania Luigi Vanvitelli, 80100 Naples, Italy; francesca.futura.bernardi@gmail.com; 6 AFO Medicina, P.O. Santa Maria delle Grazie, ASL Napoli 2 Nord, 80078 Pozzuoli, Italy

**Keywords:** low-molecular-weight heparins, enoxaparin, venous thromboembolism, acute coronary syndrome

## Abstract

Low-molecular-weight heparins are a class of drugs derived from the enzymatic depolymerization of unfractionated heparin that includes enoxaparin. Several studies have been performed on enoxaparin in recent years, in particular for the prevention and treatment of venous thromboembolism and for the treatment of acute coronary syndrome. Furthermore, the use of enoxaparin has been extended to other clinical situations that require antithrombotic pharmacological prevention, such as hemodialysis and recurrent abortion. In this review, we report the main clinical experiences of using enoxaparin in the prevention of VTE in nonsurgical patients.

## 1. Introduction

Low-molecular-weight heparins (LMWHs) are a class of anticoagulants derived from unfractionated heparin (UFH) by chemical or enzymatic depolymerization procedures [1]. In recent years, enoxaparin has been one of the most used low-molecular-weight heparins in the daily clinical management of several conditions. From a biochemical point of view, enoxaparin is a low-molecular-weight heparin obtained from porcine intestinal mucosa, and it is able to inhibit the action of several proteases involved in the clotting cascade [2]. It is an acidic mucopolysaccharide formed of equal parts sulfated D-glucosamine and D-glucuronic acid with sulfamic bridges. Its molecular weight ranges from 3800 to 5000 Daltons (mean molecular weight of approximately 4500 Daltons) [3]. Enoxaparin acts via binding antithrombin III, accelerating its action toward active proteases of the clotting system (i.e., factor IIa, factor Xa and so on). By activating antithrombin III, enoxaparin, in fact, potentiates the inhibition of factor Xa (which catalyzes the conversion of prothrombin to thrombin) and IIa (preventing the conversion of fibrinogen to fibrin) [4].

Enoxaparin has many fields of application, in particular for the prevention of venous thromboembolism (VTE) in surgical or in medical patients and for the treatment of VTE (i.e., deep-vein thrombosis and/or pulmonary embolism) [5,6,7]. Yet, it has also been used in the antithrombotic treatment of acute coronary syndrome (administered with antiplatelet drugs), including in patients managed medically or with subsequent percutaneous coronary intervention (PCI) or for prevention of thrombosis during hemodialysis [8,9,10,11].

In this review, we report the main clinical indications of the daily use of enoxaparin in nonsurgical patients.

### 1.1. Prophylaxis of Venous Thromboembolism

Deep venous thrombosis (DVT) and pulmonary embolism (PE) are the main causes of preventable death among hospitalized patients during hospitalization or after discharge [12,13]. The most relevant risk factors for VTE are major surgical interventions, serious traumas, reduced mobility, active cancer, personal history of previous VTE, elderly age, ongoing hormonal treatment, pregnancy and postpartum, thrombophilic disorders, acute medical illness such as heart failure or respiratory failures, acute myocardial infarction, ischemic stroke and acute infections [14,15]. Therefore, primary prevention strategies can be particularly useful in patients carrying thrombotic risk factors. For these reasons, strategies to prevent VTE events have been hypothesized since the 1990s to the present day, with several protocols being developed. Thromboprophylaxis can be divided into mechanical and pharmacological methods [16,17]. The former includes the early mobilization of patients, intermittent pneumatic compression of the lower limbs (IPC), venous foot pump (VFP) and graduated compression stockings (GCSs). Recommended drugs include unfractionated heparin (UFH) at a low dosage, low-molecular-weight heparins (LMWHs), fondaparinux, anticoagulant agents (VKAs) and direct oral anticoagulants (DOACs) (i.e., dabigatran, rivaroxaban, apixaban) and acetylsalicylic acid (in selected clinical conditions).

The American College of Chest Physicians (ACCP) guidelines (2012) and the American Society of Hematology (ASH) guidelines (2018) [18,19] divided VTE thromboprophylaxis into three categories, with different levels of thromboembolic and hemorrhagic risk: (1) nonsurgical patients; (2) nonorthopedic surgical patients; and (3) orthopedic surgical patients. For acutely ill hospitalized medical patients at increased risk of thrombosis, guidelines recommend anticoagulant thromboprophylaxis with low-molecular-weight heparins once daily, low-dose unfractionated heparin (LDUH) twice daily or fondaparinux once daily (Grade 1B) and suggest against extending the duration of thromboprophylaxis beyond the period of patient hypomobility after hospital discharge (Grade 2B). For acutely ill hospitalized medical patients at low risk of thrombosis, they recommend against the use of pharmacologic prophylaxis or mechanical prophylaxis (Grade 1B) [18,19]. The updated 2020 ACCP guidelines do not suggest the use of DOACs or VKAs for prophylaxis of VTE in medical patients or in acute illness.

Several studies are available in the literature regarding the above, in particular regarding the efficacy and safety of LMWHs. THE-PRINCE trial (Thromboembolism-Prevention in Cardiopulmonary Diseases with Enoxaparin) underlined the efficacy and safety of enoxaparin (40 mg once daily) compared with unfractionated heparin (5000 IU three times daily) for the prevention of venous thromboembolic disease in patients with heart failure or severe respiratory disease. Enoxaparin was at least as effective as unfractionated heparin in the prevention of thromboembolic events, with significantly fewer adverse events [20]. Furthermore, the dosages of preventive enoxaparin administered to medical patients underwent assessment through dedicated trials such as MEDENOX (MEDical patients with ENOXaparin) study. This trial not only examined the suitable dosage for thromboprophylaxis in nonsurgical patients but also provided insights into this matter. It was a double-blind, randomized, placebo-controlled trial undertaken to evaluate the efficacy of two dosage regimens of enoxaparin (40 vs. 20 mg) for the prevention of venous thromboembolism in acutely ill medical patients. The primary outcome was venous thromboembolism between days 1 and 14. The trial showed a significant reduction in venous thromboembolic events in the group treated with 40 mg daily compared with the 20 mg group and the placebo group [6]. These data on safe and effective thromboprophylaxis with enoxaparin were confirmed also in a further study in acute medical illness [21].

Regarding the duration of thromboprophylaxis after discharge, the indication for enoxaparin and other anticoagulants after hospital discharge derives from data reported in the study EXCLAIM (EXtended CLinical prophylaxis in Acutely Ill Medical patients). The EXCLAIM trial compared the efficacy and safety of extended-duration thromboprophylaxis (enoxaparin 40 mg once daily for 28 +/− 4 days) with the standard regimen (enoxaparin 40 mg once daily for 10 +/− 4 days) in acutely ill medical patients with recent reduced mobility after hospital discharge. The 28 +/− 4 days regimen demonstrated a significantly reduced incidence of venous thromboembolism and symptomatic VTE (deep-vein thrombosis or pulmonary embolism). The incidence of major bleeding episodes was higher during the extended-duration prophylaxis phase, but the rates of total events were low, without any difference in terms of mortality from bleeding in both groups [22].

### 1.2. VTE Prophylaxis in Oncological Patients

The double association between cancer and thrombosis has been well known since the first medical report in the 19th century. For this reason, several trials have been conducted in oncological patients at the beginning of this millennium using several types of LMWHs.

The CLOT study and the CATCH study underlined the reduction in morbidity and mortality for pulmonary embolism in patients taking LMWHs [23,24]. Yet, since these studies were published, an increased risk of major bleeding has been reported in particular for gastrointestinal bleeding vs. placebos. Subgroups of analysis of studies in medical patients conducted with enoxaparin confirmed the benefits of this class action, so enoxaparin is frequently used in oncological patients [25].

Yet, with the improvements in oncological care in the last few years, the natural history of cancer has similarly improved, and for this reason, several editions of the ACCP guidelines suggest the use of LMWHs for prophylaxis and the treatment of VTE in oncological patients. In the last edition of the ACCP guidelines, a specific difference was noted for thromboprophylaxis. Thromboprophylaxis, in fact, is suggested in cases of acute overlapping illness and for recent surgery or hypomobility in oncological patients, while for outpatients, routine thromboprophylaxis is suggested during chemotherapy according to the Khorana score and for patients carrying venous catheters according to the Michigan score. In any case, the use of pharmacological thromboprophylaxis is suggested when there is no bleeding risk or active bleeding.

DOACs or warfarin are also suggested for the treatment of VTE in oncological patients.

### 1.3. VTE Prophylaxis in Inpatients with Ischemic Stroke

The acute treatment of ischemic stroke when possible is indicated for thrombolysis (mechanical or pharmacological thrombolysis). Yet, for patients for whom thrombolysis is not indicated for whatever reason, other medical treatments are suggested, which are mainly based on antiplatelets with an indication for oral anticoagulation if associated with cardiac arrhythmic diseases.

In other cases for which oral anticoagulation is not indicated, pharmacological thromboprophylaxis is suggested, and the use of the PADUA score may help to identify this.

As for other medical illnesses, the use of LMWHs or UFH for VTE prophylaxis is also advised in the case of acute ischemic stroke. The efficacy and safety of enoxaparin was compared with that of unfractionated heparin in the PREVAIL (PREvention of VTE after Acute Ischemic stroke with LMWH enoxaparin) study [26]. In this study, patients with acute ischemic stroke who were unable to escape hypomobility were randomly assigned to receive either enoxaparin 40 mg subcutaneously once daily or unfractionated heparin 5000 U subcutaneously every 12 h for 10 days. The trial showed enoxaparin is preferable, reducing the risk of venous thromboembolism by 43% compared with unfractionated heparin, with a similar occurrence of any type of bleeding.

Of course, according to data previously reported in the EXCLAIM trial, pharmacological thromboprophylaxis may also be prolonged after the acute phase of ischemic stroke after discharge if hypomobility or other thrombotic risk factors are still present (i.e., a regimen of 38 days +/− 4 days of prophylaxis was safer and more effective than 10 +/− 4 days). These data are relevant because patients after ischemic stroke are usually administered combined antiplatelet treatment.

### 1.4. VTE Prophylaxis in Acute Coronary Syndrome with or without PCI

Being an acute medical emergency, acute coronary syndrome (ACS) puts patients at risk of developing complications such as pulmonary embolism. For this reason, it is included as a prothrombotic risk factor in the PADUA score in order to flag the possibility of pharmacological thromboprophylaxis in inpatients treated for ACS. Furthermore, the current literature also supports the use of enoxaparin in the management of acute coronary syndrome per se because it may improve the outcome of ACS [27,28]. In several reports, enoxaparin and other LMWHs showed good efficacy and safety versus UHF and as well as a placebo. For these reasons, LMWHs play an important role in the treatment of STEMI, in association with treatment for coronary reperfusion (intravenous thrombolysis or percutaneous coronary intervention). The ASSENT-3 study compared the efficacy and safety of Tenecteplase plus enoxaparin (or abciximab) vs. Tenecteplase plus UFH in patients with acute myocardial infarction (AMI) with onset of symptoms within 6 h. The results showed that enoxaparin or abciximab regimens reduced the frequency of ischemic complications [29]. For this reason, administration of enoxaparin was considered to be better than that of UFH when thrombolytic therapy is required [30]. The validation of this issue was provided by a larger, randomized, double-blind study, the ExTRACT-TIMI 25 (Enoxaparin and Thrombolysis Reperfusion for Acute Myocardial Infarction Treatment) study. The primary objective was to determine whether enoxaparin was superior to UFH as an adjunctive therapy in patients with acute ST-segment elevation myocardial infarction receiving fibrinolytic therapy. The trial demonstrated the superiority of enoxaparin over UFH in reducing death and recurrence of non-fatal myocardial infarction at 30 days (the primary efficacy endpoint), although this benefit was associated with an increase in major bleeding episodes [31].

These results were also confirmed by trials in which patients with ACS were treated with PCI. STEEPLE [32] (SafeTy and Efficacy of Enoxaparin in Percutaneous coronary intervention patients, an internationaL randomized Evaluation) was a randomized open-label trial undertaken to assess the safety of enoxaparin as compared with unfractionated heparin in elective PCI. The primary endpoint was the incidence of major or minor bleeding at 48 h after index PCI (excluding bypass graft bleeding). The main secondary endpoint was the achievement of therapeutic anticoagulation. A single intravenous bolus of enoxaparin 0.5 mg/kg and 0.75 mg/kg was compared with intravenous unfractionated heparin (adjusted for activated clotting time), and the superior safety profile of enoxaparin injection was demonstrated. It was associated with reduced major and minor bleeding. A better reduction in bleeding was obtained with a dose of 0.5 mg per kilogram, while the 0.75 mg/kg regimen was non-inferior to UFH (6.5% vs. 8.5%). The study also showed that enoxaparin was associated with a major rate of patients achieving target anticoagulation levels compared with UFH.

The ESSENCE (the Efficacy and Safety of Subcutaneous Enoxaparin in Non-Q-Wave Coronary Events) trial [33] made a comparison between enoxaparin and unfractionated heparin in patients with angina at rest or non-Q-wave myocardial infarction. Patients received either 100 IU of enoxaparin per kilogram of body weight subcutaneously every 12 h or continuous intravenous unfractionated heparin for a minimum of 48 h to a maximum of 8 days. All patients received 100 mg to 325 mg of acetylsalicylic acid daily too. The primary endpoint was the incidence of recurrent angina, myocardial infarction or death. The results showed a significant reduction in clinical events in the enoxaparin group compared with the UFH group after 14 days, which remained significant after 30 days. The need for coronary revascularization procedures at 30 days was also significantly lessened in the patients assigned to enoxaparin.

In this clinical setting, data provided by the reported studies underlined that the administration of enoxaparin in ACS is a relevant part of the complex antithrombotic therapy based on antiplatelet treatment (e.g., single-antiplatelet treatment or dual-antiplatelet treatment) and is associated with an improved outcome of the disease.

Enoxaparin did not exhibit statistical significance compared with UFH only in the trial in which inpatients with ACS showed non-ST segment elevation MI. The SYNERGY trial, in fact, showed the superiority of enoxaparin over UFH in high-risk, non-ST-segment elevation MI (NSTEMI); there were no significant differences in the rates of death, stroke or MI at 30 days between the two groups [34].

### 1.5. VTE Prophylaxis in Obstetric Conditions

VTE is one of the most dangerous complications during pregnancy because it is associated with increased morbidity and mortality of pregnant women. Pregnancy, in fact, is per se a thrombotic risk factor and VTE in pregnant women usually occurs because of the presence of several further thrombotic risk factors such as hypomobility, thrombophilia and hormonal therapy during pregnancy. A prothrombotic state is present during pregnancy, and this acquired prothrombotic condition does not end with delivery; VTE, in fact, is also strongly associated with puerperium. For this reason, thromboprophylaxis with enoxaparin has been stated to be safe for the prevention of VTE in pregnancy in several cohorts and/or guidelines [35,36]. Moreover, in the case of overt VTE during pregnancy or puerperium, enoxaparin is considered in the same guidelines as the first choice of treatment for VTE in acute and subacute phases of diseases.

Intriguingly, the reported prothrombotic risk factors are associated with pregnancy not only with an increased incidence of VTE but also with recurrent pregnancy loss or pregnancy adverse outcomes (i.e., preterm delivery, HELLP syndrome, pre-eclampsia). Yet, in the obstetric field, there are more cohorts and trials available regarding the safe use of enoxaparin and the improved obstetric outcome (i.e., live births with a reduction in miscarriage or pre-eclampsia/growth reduction) [37].

For these reasons, enoxaparin has been determined to be a safe drug in women at risk of miscarriage or other obstetric complications as far as those at increased risk of VTE, as judged by experts and guidelines.

### 1.6. VTE Prophylaxis in Patients with Cava Filters

Chronic obstruction of the ileo-caval veins can be caused by many conditions with inflammatory, compressive or intraluminal causes. With the aim of the restoration of venous flow to prevent and treat post-thrombotic syndrome and reduce pulmonary embolism occurrence, chronic obstruction of the IVC has been historically treated with the ligation of infra-renal IVC first and then with IVC filters [38]. Modifications in the hemodynamics of the inferior vena cava (IVC) following the insertion of a filter could potentially elevate the likelihood of thrombosis [39]. Furthermore, available evidence indicates that IVC filters amplify the risk of deep-vein thrombosis (DVT) [40]. As a result, it is advisable to initiate aggressive anticoagulation therapy with enoxaparin promptly upon IVC filter placement followed by concurrent administration of a vitamin K antagonist (VKA) [41,42]. The therapeutic goal is to achieve an international normalized ratio (INR) within the range of 2.0–3.0. Research findings have solidified the affirmation that low-molecular-weight heparin is both safe and efficacious in prophylaxis in this clinical setting; furthermore, the use of LMWHs in this clinical setting has been demonstrated to be effective for patients with cancer or pregnant patients [43,44,45].

### 1.7. Other Medical Conditions in which Prophylaxis with Enoxaparin Is Frequently Used

The use of prophylactic doses of LMWHs is still suggested in several medical illnesses, such as inflammatory bowel diseases (IBDs), kidney failure and female infertility.

Yet, although the use of LMWHs in several abdominal diseases is accepted, specific guidelines are still lacking in particular for IBDs. IBDs, in fact, are frequently associated with thrombotic events, and pharmacological prophylaxis with LMWHs is possible during acute phases of inflammation, according to the PADUA score [46]. Yet, during chronic phases of inflammation, antithrombotic prophylaxis is not suggested by international guidelines, and in the majority of cases, this suggestion is dependent on the occurrence of bleeding events, which are also frequent in the natural history of IBDs.

Furthermore, LMWHs are the golden standard for the acute treatment of abdominal vein thrombosis (AVT), including portal vein thrombosis secondary to liver cirrhosis [47]. Yet, although LMWHs and in particular enoxaparin have been suggested by guidelines for the treatment of AVT, there are no specific suggestions for the prevention of these thrombotic complications with prophylactic doses of LMWHs.

On the other hand, VTE is one of the most common complications in patients with intracranial hemorrhage [48]. Therefore, after acute bleeding ends, thromboprophylaxis is usually suggested in any case. LMWHs have been used in several studies without an increase in fatal bleeding, although specific guidelines are not present [49]. In these cases, in fact, international guidelines suggest intermittent pneumatic compression as a priority as opposed to LMWHs.

Moreover, the use of enoxaparin in patients undergoing in vitro fertilization procedures (IVFPs) is frequently suggested by gynecologists. Two different ways of administering LMWHs are present in this field: prophylaxis of VTE in women at risk for the presence of thrombotic risk factors and good outcomes of IVFPs regarding successful pregnancy. There are no guidelines suggesting the routine use of enoxaparin or other LMWHs in this field. VTE prophylaxis is not suggested for routine use because the rate of VTE events after IVFPs is very low, while the use of enoxaparin to increase the chance of a successful pregnancy is not based on the evidence of clinical trials [50].

Another extended field of application of thromboprophylaxis with enoxaparin is chronic kidney disease (CKD). Being a public health burden, CKD and its complications, such as venous thromboembolism, atherothrombosis and occlusion of the vascular access used for chronic hemodialysis, justify the use of long-term prophylaxis of thrombotic diseases in CKD. Because of renal impairment, the pharmacokinetics of several drugs are different in CKD. Reduced kidney glomerular filtration requires an adjusted dose of several drugs during the chronic treatment of chronic diseases. For this reason, direct oral anticoagulants are not suggested when glomerular filtration is reduced, while administration of LMWHs or fondaparinux is possible with adjusted doses.

Guidelines for pharmacological thromboprophylaxis suggest regular doses of LMWHs for prophylaxis in stable CKD, while in the case of glomerular filtrates, less than 20 mL/h and the occurrence of a VTE, the suggested dose of enoxaparin is 1 mg/kg once daily [51,52]. For patients with end-stage renal diseases for whom hemodialysis is performed routinely, long-term VTE prophylaxis with LMWHs or with enoxaparin is not suggested by guidelines. The paucity of literature showing long-term outcomes in this field and the frequent complications, such as major bleeding, in this setting do not permit long-term administration of enoxaparin or other LMWHs [53].

### 1.8. Other Potential Effects of Enoxaparin in Daily Treatment of Medical Illness

The pleiotropic effects of heparins have been discussed for several years. The antiviral, anti-inflammatory and antimetastatic actions of heparins were already recognized several years ago in vitro, but their actions in vivo have not been confirmed.

The formation of a cancer-cell-encircling platelet cloak which facilitates metastasis is well known, and heparins, with their ligand with p-selectin, inhibit these activities in vitro, as demonstrated with spectroscopy and other experimental models in particular on melanoma cell lines [54,55,56].

Although these properties of heparins are well recognized, determining the therapeutic dose of heparin needed to inhibit metastasis is very difficult. and for this reason. large trials are still needed.

Yet, recently, clinical experiences during the pandemic caused by SARS-CoV-2 permitted a better understanding of the anti-inflammatory actions of heparins besides its use as an anticoagulant. Being a protease inhibitor, heparins also have an anti-inflammatory pleiotropic effect, with improved outcomes being shown for several cohorts of patients treated with different doses of enoxaparin or fondaparinux during the pandemic [57,58,59].

Furthermore, in vitro models have demonstrated that heparins are able to bind SARS-CoV-2. Heparin, in fact, has a specific ability similar to that of other oligosaccharides and glycosaminoglycan to bind several types of viruses as they pass through the extracellular matrix of the respiratory tract, thus reducing the overload and cytolysis of the respiratory tract [60]. The improved outcomes of inpatients treated with increased doses of heparins during the pandemic are also probably due to this pleiotropic effect.

## 2. Thromboprophylaxis in Surgical Patients

We should underline that the use of prophylaxis with enoxaparin for inpatients began with surgical patients to prevent post-operative VTE. In the last few years, a lot of studies and trials have demonstrated that several LMWHs are safe and effective in reducing the rate of post-operative VTE [61].

Enoxaparin showed its efficacy and safety in different trials, such as the ENOXACAN and ENOXACAN II studies, which showed the efficacy of prophylactic doses of enoxaparin for 4 weeks after abdominal oncological surgery vs. a placebo in the reduction in VTE in this clinical setting [62].

Of course, several further studies were performed in this setting, showing the same efficacy, in particular in abdominal surgery [63].

Similarly, extended thromboprophylaxis with enoxaparin once daily for 4 weeks after surgery was demonstrated to be effective after major orthopedic surgery at the beginning of the new millennium, as reported in different studies [64,65].

Along with this prolonged and certified scientific exploration in different clinical settings in patients with acute surgical and medical illnesses, enoxaparin was also tested in other studies as a golden standard to prevent primary or secondary VTE and compared with other anticoagulants, such as direct oral anticoagulants [66,67,68,69,70,71,72,73].

However, subgroups for which enoxaparin or other LMWHs remain valid support for thromboprophylaxis are summarized in Table 1. The table offers a clinical scenario of international opinions on the use of enoxaparin or other LMWHs in inpatients in medical or surgical areas. Herein, we can see enoxaparin remains one of the most used anticoagulant drugs. It remains a golden standard for oncological patients during hospitalization and also during chemotherapies for patients with acute illness, infectious diseases (e.g., COVID-19 or bacterial sepsis) and acute thrombotic events of arterial/venous vessels; in these areas, enoxaparin has proven a high-profile drug, with its efficacy and safety being demonstrated by several studies conducted over 25 years.

In the same way, inpatients in surgical areas found clinical advantages from the use of LMWHs, and in these fields, enoxaparin’s efficacy and safety have been proven by several studies in orthopedics and abdominal surgery.

## 3. Conclusions

In conclusion, a multitude of inquiries have meticulously examined the utility, efficacy and safety of enoxaparin in thromboprophylaxis for venous thromboembolism among medical patients. The extensive focus of these investigations has centered on the prevention and management of venous thromboembolism, showcasing enoxaparin’s notable effectiveness. Moreover, the demonstrably positive outcomes observed in the context of patients grappling with acute coronary syndrome have been extensively chronicled in the aforementioned studies. Consequently, the contemporary landscape still testifies to the widespread use of enoxaparin in various clinical scenarios requiring pharmacologically guided antithrombotic interventions, and the recent experience of the pandemic indicated that the clinical application of enoxaparin may increase in the future.

## Figures and Tables

**Table 1:** Approved clinical conditions for which LMWHs are suggested by international guidelines.

Guideline Indication for VTE Prevention in Inpatients	**ASH**	**ASCO**	**ACCP**	**NICE**
COVID-19 inpatients with respiratory impairment	Yes	/	Yes	Yes
Acute medical illness inpatients (according to PADUA score)	Yes	/	Yes	Yes
Oncological VTE prevention in medical conditions (according to Khorana score or Michigan score)	Yes	Yes	Yes	Yes
Oncological surgery	Yes	Yes	Yes	Yes
Major ortohpaedic surgery	Yes	/	Yes	Yes
Traditional orthopaedic surgery	Yes	/	Yes	Yes

VTE, venous thromboembolism; ASH, American Society of Haematology; ASCO, American Society of Clinical Oncology; ACCP, American College of Chest Physicians; NICE, National Institute for Health and Care Excellence.

## References

[1] Hirsh J. (1998). Low-molecular-weight heparin: A review of the results of recent studies of the treatment of venous thromboembolism and unstable angina. Circulation.

[2] Hogwood J., Mulloy B., Lever R., Gray E., Page C.P. (2023). Pharmacology of Heparin and Related Drugs: An Update. Pharmacol. Rev..

[3] Iqbal Z., Cohen M. (2011). Enoxaparin: A pharmacologic and clinical review. Expert. Opin. Pharmacother..

[4] Fareed J., Hoppensteadt D., Walenga J., Iqbal O., Ma Q., Jeske W., Sheikh T. (2003). Pharmacodynamic and pharmacokinetic properties of enoxaparin: Implications for clinical practice. Clin. Pharmacokinet..

[5] Warner G.T., Perry C.M. (2001). Enoxaparin: In the prevention of venous thromboembolism in medical patients. Am. J. Cardiovasc. Drugs.

[6] Turpie A.G. (2000). Thrombosis prophylaxis in the acutely ill medical patient: Insights from the prophylaxis in MEDical patients with ENOXaparin (MEDENOX) trial. Am. J. Cardiol..

[7] Lamy A., Wang X., Kent R., Smith K.M., Gafni A. (2002). Economic evaluation of the MEDENOX trial: A Canadian perspective. Medical Patients with Enoxaparin. Can. Respir. J..

[8] Vareesangthip K., Thitiarchakul S., Kanjanakul I., Sumethkul V., Krairittichai U., Chittinandana A., Bannachak D. (2011). Efficacy and safety of enoxaparin during hemodialysis: Results from the HENOX study. J. Med. Assoc. Thai..

[9] Santos A., Macías N., Vega A., Abad S., Linares T., Aragoncillo I., Cruzado L., Pascual C., Goicoechea M., López-Gómez J.M. (2020). Efficacy of enoxaparin in preventing coagulation during high-flux haemodialysis, expanded haemodialysis and haemodiafiltration. Clin. Kidney J..

[10] Wiegele M., Adelmann D., Dibiasi C., Pausch A., Baierl A., Schaden E. (2021). Monitoring of Enoxaparin during Hemodialysis Covered by Regional Citrate Anticoagulation in Acute Kidney Injury: A Prospective Cohort Study. J. Clin. Med..

[11] Lee S., Gibson C.M. (2007). Enoxaparin in acute coronary syndromes. Expert. Rev. Cardiovasc. Ther..

[12] Konstantinides S.V., Meyer G., Becattini C., Bueno H., Geersing G.J., Harjola V.P., Huisman M.V., Humbert M., Jennings C.S., Jiménez D. (2020). 2019 ESC Guidelines for the diagnosis and management of acute pulmonary embolism developed in collaboration with the European Respiratory Society (ERS). Eur. Heart J..

[13] Henke P.K., Kahn S.R., Pannucci C.J., Secemksy E.A., Evans N.S., Khorana A.A., Creager M.A., Pradhan A.D., American Heart Association Advocacy Coordinating Committee (2020). Call to Action to Prevent Venous Thromboembolism in Hospitalized Patients: A Policy Statement From the American Heart Association. Circulation.

[14] Heit J.A. (2015). Epidemiology of venous thromboembolism. Nat. Rev. Cardiol..

[15] Neeman E., Liu V., Mishra P., Thai K.K., Xu J., Clancy H.A., Schlessinger D., Liu R. (2022). Trends and Risk Factors for Venous Thromboembolism Among Hospitalized Medical Patients. JAMA Netw. Open..

[16] Park J., Lee J.M., Lee J.S., Cho Y.J. (2016). Pharmacological and Mechanical Thromboprophylaxis in Critically Ill Patients: A Network Meta-Analysis of 12 Trials. J. Korean Med. Sci..

[17] Turner B.R.H., Machin M., Jasionowska S., Salim S., Onida S., Shalhoub J., Davies A.H. (2023). Systematic Review and Meta-analysis of the Additional Benefit of Pharmacological Thromboprophylaxis for Endovenous Varicose Vein Interventions. Ann. Surg..

[18] Schünemann H.J., Cushman M., Burnett A.E., Kahn S.R., Beyer-Westendorf J., Spencer F.A., Rezende S.M., Zakai N.A., Bauer K.A., Dentali F. (2018). American Society of Hematology 2018 guidelines for management of venous thromboembolism: Prophylaxis for hospitalized and nonhospitalized medical patients. Blood Adv..

[19] Guyatt G.H., Akl E.A., Crowther M., Gutterman D.D., Schuünemann H.J., American College of Chest Physicians Antithrombotic Therapy and Prevention of Thrombosis Panel (2012). Executive summary: Antithrombotic Therapy and Prevention of Thrombosis, 9th ed: American College of Chest Physicians Evidence-Based Clinical Practice Guidelines. Chest.

[20] Kleber F.X., Witt C., Vogel G., Koppenhagen K., Schomaker U., Flosbach C.W., THE-PRINCE Study Group (2003). Randomized comparison of enoxaparin with unfractionated heparin for the prevention of venous thromboembolism in medical patients with heart failure or severe respiratory disease. Am. Heart J..

[21] Kakkar A.K., Cimminiello C., Goldhaber S.Z., Parakh R., Wang C., Bergmann J.F., LIFENOX Investigators (2011). Low-molecular-weight heparin and mortality in acutely ill medical patients. N. Engl. J. Med..

[22] Hull R.D., Schellong S.M., Tapson V.F., Monreal M., Samama M.M., Turpie A.G., Wildgoose P., Yusen R.D. (2006). Extended-duration thromboprophylaxis in acutely ill medical patients with recent reduced mobility: Methodology for the EXCLAIM study. J. Thromb. Thrombolysis.

[23] Lee A.Y., Levine M.N., Baker R.I., Bowden C., Kakkar A.K., Prins M., Rickles F.R., Julian J.A., Haley S., Kovacs M.J. (2003). Randomized Comparison of Low-Molecular-Weight Heparin versus Oral Anticoagulant Therapy for the Prevention of Recurrent Venous Thromboembolism in Patients with Cancer (CLOT) Investigators. Low-molecular-weight heparin versus a coumarin for the prevention of recurrent venous thromboembolism in patients with cancer. N. Engl. J. Med..

[24] Lee A.Y., Bauersachs R., Janas M.S., Jarner M.F., Kamphuisen P.W., Meyer G., Khorana A.A., CATCH Investigators (2013). CATCH: A randomised clinical trial comparing long-term tinzaparin versus warfarin for treatment of acute venous thromboembolism in cancer patients. BMC Cancer.

[25] Diener H.C., Foerch C., Riess H., Röther J., Schroth G., Weber R. (2013). Treatment of acute ischaemic stroke with thrombolysis or thrombectomy in patients receiving anti-thrombotic treatment. Lancet Neurol..

[26] Evans C.P., Higano C.S., Keane T., Andriole G., Saad F., Iversen P., Miller K., Kim C.S., Kimura G., Armstrong A.J. (2016). The PREVAIL Study: Primary Outcomes by Site and Extent of Baseline Disease for Enzalutamide-treated Men with Chemotherapy-naïve Metastatic Castration-resistant Prostate Cancer. Eur. Urol..

[27] Antman E.M., McCabe C.H., Gurfinkel E.P., Turpie A.G., Bernink P.J., Salein D., Bayes De Luna A., Fox K., Lablanche J.M., Radley D. (1999). Enoxaparin prevents death and cardiac ischemic events in unstable angina/non-Q-wave myocardial infarction. Results of the thrombolysis in myocardial infarction (TIMI) 11B trial. Circulation.

[28] Schmidt-Lucke C., Schultheiss H.P. (2007). Enoxaparin injection for the treatment of high-risk patients with non-ST elevation acute coronary syndrome. Vasc. Health Risk Manag..

[29] Assessment of the Safety and Efficacy of a New Thrombolytic Regimen (ASSENT)-3 Investigators (2001). Efficacy and safety of tenecteplase in combination with enoxaparin, abciximab, or unfractionated heparin: The ASSENT-3 randomised trial in acute myocardial infarction. Lancet.

[30] El-Rayes M., Schampaert E., Tardif J.C., Eisenberg M.J., Afilalo M., Kouz S., Lauzon C., Harvey R., Nguyen M., Kouz R. (2010). Safety and effectiveness of enoxaparin following fibrinolytic therapy: Results of the Acute Myocardial Infarction (AMI)-QUEBEC registry. Can. J. Cardiol..

[31] Gabriel R.S., White H.D. (2007). ExTRACT-TIMI 25 trial: Clarifying the role of enoxaparin in patients with ST-elevation myocardial infarction receiving fibrinolysis. Expert. Rev. Cardiovasc. Ther..

[32] Montalescot G., White H.D., Gallo R., Cohen M., Steg P.G., Aylward P.E., Bode C., Chiariello M., King SB 3rd Harrington R.A., Desmet W.J. (2006). Enoxaparin versus unfractionated heparin in elective percutaneous coronary intervention. N. Engl. J. Med..

[33] Cohen M., Blaber R., Demers C., Gurfinkel E.P., Langer A., Fromell G., Premmereur J., Turpie A.G. (1997). The Essence Trial: Efficacy and Safety of Subcutaneous Enoxaparin in Unstable Angina and Non-Q-Wave MI: A Double-Blind, Randomized, Parallel-Group, Multicenter Study Comparing Enoxaparin and Intravenous Unfractionated Heparin: Methods and Design. J. Thromb. Thrombolysis.

[34] SYNERGY Executive Committee (2002). Superior Yield of the New strategy of Enoxaparin, Revascularization and GlYcoprotein IIb/IIIa inhibitors. The SYNERGY trial: Study design and rationale. Am. Heart J..

[35] Greer I.A., Nelson-Piercy C. (2005). Low-molecular-weight heparins for thromboprophylaxis and treatment of venous thromboembolism in pregnancy: A systematic review of safety and efficacy. Blood.

[36] American College of Obstetricians and Gynecologists’ Committee on Practice Bulletins—Obstetrics (2018). ACOG Practice Bulletin No. 196: Thromboembolism in Pregnancy. Obstet. Gynecol..

[37] Shirazi M., Naeiji Z., Sharbaf F.R., Golshahi F., Fathi M., Nazari F., Sahebdel B. (2021). Therapeutic role of enoxaparin in intra-uterine growth restriction: A randomized clinical trial. J. Gynecol. Obstet. Hum. Reprod..

[38] Wehrenberg-Klee E., Stavropoulos S.W. (2012). Inferior vena cava filters for primary prophylaxis: When are they indicated?. Semin. Intervent Radiol..

[39] Singer M.A., Wang S.L. (2011). Modeling blood flow in a tilted inferior vena cava filter: Does tilt adversely affect hemodynamics?. J. Vasc. Interv. Radiol..

[40] Sarosiek S., Crowther M., Sloan J.M. (2013). Indications, complications, and management of inferior vena cava filters: The experience in 952 patients at an academic hospital with a level I trauma center. JAMA Intern. Med..

[41] Kearon C., Akl E.A., Ornelas J., Blaivas A., Jimenez D., Bounameaux H., Huisman M., King C.S., Morris T.A., Sood N. (2016). Antithrombotic Therapy for VTE Disease: CHEST Guideline and Expert Panel Report. Chest.

[42] Jain A., Cifu A.S. (2017). Antithrombotic Therapy for Venous Thromboembolic Disease. JAMA.

[43] Breddin H.K., Hach-Wunderle V., Nakov R., Kakkar V.V., CORTES Investigators (2001). Clivarin: Assessment of Regression of Thrombosis, Efficacy, and Safety. Effects of a low-molecular-weight heparin on thrombus regression and recurrent thromboembolism in patients with deep-vein thrombosis. N. Engl. J. Med..

[44] Voigtlaender M., Langer F. (2019). Low-Molecular-Weight Heparin in Cancer Patients: Overview and Indications. Hamostaseologie.

[45] Mismetti P., Laporte S., Pellerin O., Ennezat P.V., Couturaud F., Elias A., Falvo N., Meneveau N., Quere I., Roy P.M. (2015). Effect of a retrievable inferior vena cava filter plus anticoagulation vs anticoagulation alone on risk of recurrent pulmonary embolism: A randomized clinical trial. JAMA.

[46] Olivera P.A., Zuily S., Kotze P.G., Regnault V., Al Awadhi S., Bossuyt P., Gearry R.B., Ghosh S., Kobayashi T., Lacolley P. (2021). International consensus on the prevention of venous and arterial thrombotic events in patients with inflammatory bowel disease. Nat. Rev. Gastroenterol. Hepatol..

[47] Shukla A., Giri S. (2022). Portal Vein Thrombosis in Cirrhosis. J. Clin. Exp. Hepatol..

[48] Cai Q., Zhang X., Chen H. (2021). Patients with venous thromboembolism after spontaneous intracerebral hemorrhage: A review. Thromb. J..

[49] Ianosi B., Gaasch M., Rass V., Huber L., Hackl W., Kofler M., Schiefecker A.J., Addis A., Beer R., Rhomberg P. (2019). Early thrombosis prophylaxis with enoxaparin is not associated with hematoma expansion in patients with spontaneous intracerebral hemorrhage. Eur. J. Neurol..

[50] Di Micco P., Russo V., Mastroiacovo D., Bosevski M., Lodigiani C. (2020). In vitro Fertilization Procedures with Embryo Transfer and Their Association with Thrombophilia, Thrombosis and Early Antithrombotic Treatments. J. Blood Med..

[51] Prophylaxis and Treatment of VTE in Renal Impairment Approved September 2020 and Reviewed in September 2023 by Drugs and Therapeutics Committee. https://gloshospitals.nhs.uk.

[52] Capodanno D., Angiolillo D.J. (2012). Antithrombotic therapy in patients with chronic kidney disease. Circulation.

[53] Sacks J., Luc S.A. (2021). Evaluation of Enoxaparin for Inpatient Venous Thromboembolism Prophylaxis in End-Stage Renal Disease Patients on Hemodialysis. Hosp. Pharm..

[54] Hostettler N., Naggi A., Torri G., Ishai-Michaeli R., Casu B., Vlodavsky I., Borsig L. (2007). P-selectin- and heparanase-dependent antimetastatic activity of non-anticoagulant heparins. FASEB J..

[55] Liebsch A.G., Schillers H. (2021). Quantification of heparin’s antimetastatic effect by single-cell force spectroscopy. J. Mol. Recognit..

[56] Kenessey I., Simon E., Futosi K., Bereczky B., Kiss A., Erdödi F., Gallagher J.T., Tímár J., Tóvári J. (2009). Antimigratory and antimetastatic effect of heparin-derived 4-18 unit oligosaccharides in a preclinical human melanoma metastasis model. Thromb. Haemost..

[57] Cardillo G., Viggiano G.V., Russo V., Mangiacapra S., Cavalli A., Castaldo G., Agrusta F., Bellizzi A., Amitrano M., Iannuzzo M. (2021). Antithrombotic and Anti-Inflammatory Effects of Fondaparinux and Enoxaparin in Hospitalized COVID-19 Patients: The FONDENOXAVID Study. J. Blood Med..

[58] Russo V., Cardillo G., Viggiano G.V., Mangiacapra S., Cavalli A., Fontanella A., Agrusta F., Bellizzi A., Amitrano M., Iannuzzo M. (2020). Thromboprofilaxys With Fondaparinux vs. Enoxaparin in Hospitalized COVID-19 Patients: A Multicenter Italian Observational Study. Front. Med..

[59] Di Micco P., Tufano A., Cardillo G., Imbalzano E., Amitrano M., Lodigiani C., Bellizzi A., Camporese G., Cavalli A., De Stefano C. (2021). The Impact of Risk-Adjusted Heparin Regimens on the Outcome of Patients with COVID-19 Infection. A Prospective Cohort Study. Viruses.

[60] Di Micco P., Imbalzano E., Russo V., Attena E., Mandaliti V., Orlando L., Lombardi M., Di Micco G., Camporese G., Annunziata S. (2021). Heparin and SARS-CoV-2: Multiple Pathophysiological Links. Viruses.

[61] Anderson D.R., Morgano G.P., Bennett C., Dentali F., Francis C.W., Garcia D.A., Kahn S.R., Rahman M., Rajasekhar A., Rogers F.B. (2019). American Society of Hematology 2019 guidelines for management of venous thromboembolism: Prevention of venous thromboembolism in surgical hospitalized patients. Blood Adv..

[62] Bergqvist D., Agnelli G., Cohen A.T., Eldor A., Nilsson P.E., Le Moigne-Amrani A., Dietrich-Neto F., ENOXACAN II Investigators (2002). Duration of prophylaxis against venous thromboembolism with enoxaparin after surgery for cancer. N. Engl. J. Med..

[63] Reiertsen O., Larsen S., Størkson R., Trondsen E., Løvig T., Andersen O.K., Lund H., Mowinckel P. (1993). Safety of enoxaparin and dextran-70 in the prevention of venous thromboembolism in digestive surgery. A play-the-winner-designed study. Scand. J. Gastroenterol..

[64] Comp P.C., Spiro T.E., Friedman R.J., Whitsett T.L., Johnson G.J., Gardiner GAJr Landon G.C., Jové M., Enoxaparin Clinical Trial Group (2001). Prolonged enoxaparin therapy to prevent venous thromboembolism after primary hip or knee replacement. Enoxaparin Clinical Trial Group. J. Bone Joint Surg. Am..

[65] Bergqvist D., Benoni G., Björgell O., Fredin H., Hedlundh U., Nicolas S., Nilsson P., Nylander G. (1996). Low-molecular-weight heparin (enoxaparin) as prophylaxis against venous thromboembolism after total hip replacement. N. Engl. J. Med..

[66] Kakkar A.K., Brenner B., Dahl O.E., Eriksson B.I., Mouret P., Muntz J., Soglian A.G., Pap A.F., Misselwitz F., Haas S. (2008). Extended duration rivaroxaban versus short-term enoxaparin for the prevention of venous thromboembolism after total hip arthroplasty: A double-blind, randomised controlled trial. Lancet.

[67] Turpie A.G., Lassen M.R., Davidson B.L., Bauer K.A., Gent M., Kwong L.M., Cushner F.D., Lotke P.A., Berkowitz S.D., Bandel T.J. (2009). Rivaroxaban versus enoxaparin for thromboprophylaxis after total knee arthroplasty (RECORD4): A randomised trial. Lancet.

[68] Lassen M.R., Ageno W., Borris L.C., Lieberman J.R., Rosencher N., Bandel T.J., Misselwitz F., Turpie A.G., RECORD3 Investigators (2008). Rivaroxaban versus enoxaparin for thromboprophylaxis after total knee arthroplasty. N. Engl. J. Med..

[69] Lassen M.R., Davidson B.L., Gallus A., Pineo G., Ansell J., Deitchman D. (2007). The efficacy and safety of apixaban, an oral, direct factor Xa inhibitor, as thromboprophylaxis in patients following total knee replacement. J. Thromb. Haemost..

[70] Lassen M.R., Raskob G.E., Gallus A., Pineo G., Chen D., Portman R.J. (2009). Apixaban or enoxaparin for thromboprophylaxis after knee replacement. N. Engl. J. Med..

[71] Li X.M., Sun S.G., Zhang W.D. (2012). Apixaban versus enoxaparin for thromboprophylaxis after total hip or knee arthroplasty: A meta-analysis of randomized controlled trials. Chin. Med. J..

[72] Lassen M.R., Gallus A., Raskob G.E., Pineo G., Chen D., Ramirez L.M., ADVANCE-3 Investigators (2010). Apixaban versus enoxaparin for thromboprophylaxis after hip replacement. N. Engl. J. Med..

[73] Lassen M.R., Raskob G.E., Gallus A., Pineo G., Chen D., Hornick P., ADVANCE-2 investigators (2010). Apixaban versus enoxaparin for thromboprophylaxis after knee replacement (ADVANCE-2): A randomised double-blind trial. Lancet.

